# Foreign Correspondence

**Published:** 1892-08

**Authors:** J. E. Thompson

**Affiliations:** Professor of Surgery Texas Medical College, and Surgeon to Sealy Hospital. Caledonian Hotel, Nottingham


### FOREIGN CORRESPONDENCE.

### Letter from Prof. J. E. Thompson, M. D.*

CALEDONIAN HOTEL,<br>NOTTINGHAM, July 28, 1892.

*Editor Daniel's Texas Medical Journal:*

. . I send you with the same post a copy of the programme of the British Medical Association, also Lawson Tait's results in hepatic surgery.

The subject was introduced by Mayo Robson, and he fairly took the wind out of Tait's sails. He read a splendid paper, not dealing, however, with hepatic abscess as much as with the surgery of the gall bladder. All his points of treatment I cannot give you, but the points of diagnosis were summed up as follows:

1. Gall stones may exist without any symptoms.

2. Impaction of a gall stone in the cystic duct is not accompanied by jaundice.

3. If jaundice happen to be present, the stone is either in the hepatic duct or the common bile duct.

4. The signs of impaction of a gall stone in the bile duct are: (*a*) Ague-like attacks with pain in the right hypochondriac. (*b*) Slight febrile condition, (*c*) followed by slight and transitory jaundice.

5. If persistence of jaundice is noticed, then probably malignant disease is present.

---

*Professor of Surgery Texas Medical College, and Surgeon to Sealy Hospital.

I just mention these points by the way, as they are interesting.

I leave here to-morrow for Belfast, and thence along the west coast of Ireland to the lakes of Killarney. I am having a fine time, and just at present the weather is very pleasant. To-day a rule was passed allowing women to become members of the British Medical Association.

I leave here for Texas the first week in September.

I am, yours faithfully,

J. E. Thompson.

[It will be remembered Dr. Thompson had a severe spell of typhoid fever in March and April, and was convalescing when he left for Europe. He writes that he is entirely well, and has regained his usual weight.—Ed.]

